# Nurses’ knowledge and attitudes regarding postpartum depression

**DOI:** 10.1192/j.eurpsy.2025.1311

**Published:** 2025-08-26

**Authors:** A. Chouchane, A. Gaddour, M. Bouhoula, Y. Tebourbi, A. Aloui, M. Maoua, A. Brahem, H. Kalboussi, O. El Maalel, I. Kacem, S. Chatti

**Affiliations:** 1 University of Sousse, Faculty of Medicine of Sousse, Occupational Medicine and Professional Pathologies Department, Farhat Hached Teaching Hospital, Tunisia.; 2 Occupational Medicine and Professional Pathologies Department, Farhat Hached Teaching Hospital, University of Sousse, Faculty of Medicine of Sousse, Sousse, Tunisia.; 3 University of Sousse, Faculty of Medicine of Sousse, Occupational Medicine Department- Ibn El Jazzar Hospital, Kairouan, Tunisia; 4 Occupational Medicine and Professional Pathologies Department, Farhat Hached Teaching Hospital, Tunisia., Sousse, Tunisia

## Abstract

**Introduction:**

Postpartum depression (PPD) is a serious mental health condition that can be challenging to diagnose due to its varying symptoms. As a result, multiple healthcare providers collaborate in its prevention, screening, and treatment. Nurses play a pivotal role in this process, requiring essential knowledge and skills

**Objectives:**

To assess nurses’ knowledge and attitudes regarding PPD.

**Methods:**

A cross-sectional study was conducted among maternity nurses at a university hospital who have direct contact with postpartum patients. Our sample was a convenience sampleData was collected using a self-administered anonymous questionnaire. The questionnaire consisted of two parts : the first part collected demographic information about the nurses, and the second part assessed nurses’ knowledge and attitudes regarding postpartum depression.

**Results:**

A total of 50 nurses participated in the study. Nearly half (49%) of participants had over 10 years of professional seniority. The majority (92%) were aware of the postpartum period during which the disorder occurs, as well as the early warning signs of PPD (82%). However, more than half of the participants (52%) were unaware of the risk factors for PPD. The majority (92%) were aware that nurses play a major role in early detection of this disorder. Only 52% knew that the Edinburgh Postnatal Depression Scale (EPDS) is the screening tool for PPD. Nearly half of the suggestions to improve the management of PPD (46%) focused on improving patient conditions and well-being. Moreover, 40% of participants made suggestions centered on collaboration and communication.

**Conclusions:**

Despite their involvement in postpartum care, nurses demonstrated limited knowledge of postpartum depression risk factors, emphasizing the need for enhanced education and support.

**Disclosure of Interest:**

None Declared

